# Supplementary material for: Escaping the benthos with Coral Reef Arks: effects on coral translocation and fish biomass
Source: PeerJ. 2024 Jul 25;12:e17640. doi: 10.7717/peerj.17640 (PMC11283776; doi:10.7717/peerj.17640)
Supplement: Supplemental Information 2 — The number and taxa of corals deployed to the Coral Arks and control sites. [file peerj-12-17640-s002.docx]

Supplemental table 1: Summary of deployed corals tracked for survival and growth.

| Stage | Treatment | Coral taxa | Coral source | Nubbins deployed |
| --- | --- | --- | --- | --- |
| 1 (Nov 2021) | Ark | *Acropora cervicornis*  *Porites porites*  *Porites porites*  *Siderastrea radians* | Palominos nursery  Palominos nursery  Bahia de Jobos  Bahia de Jobos | 44  7  23  21 |
|  | Control | *Acropora cervicornis*  *Porites porites*  *Porites porites*  *Siderastrea radians* | Palominos nursery  Palominos nursery  Bahia de Jobos  Bahia de Jobos | 39  8  24  26 |
| 2 (May 2022) | Ark | *Acropora cervicornis*  *Porites porites*  *Orbicella faveolata*  *Orbicella franksii*  *Agaricia* sp*.*  *Porites astreoides*  *Porites furcata*  *Siderastrea siderea* | Palominos nursery  Palominos nursery  Palominos nursery  Palominos nursery  Mosquito Pier  Mosquito Pier  Mosquito Pier  Mosquito Pier | 20  20  2  2  9  8  20  20 |
|  | Control | *Acropora cervicornis*  *Porites porites*  *Orbicella faveolata*  *Orbicella franksii*  *Agaricia* sp*.*  *Porites astreoides*  *Porites furcata*  *Siderastrea siderea* | Palominos nursery  Palominos nursery  Palominos nursery  Palominos nursery  Mosquito Pier  Mosquito Pier  Mosquito Pier  Mosquito Pier | 19  20  2  1  8  8  20  20 |
